# The Impact of the Social Mood on the Italian Sovereign Debt Market: A Twitter Perspective

**DOI:** 10.1007/s40797-022-00217-z

**Published:** 2023-01-18

**Authors:** Giovanni Carnazza

**Affiliations:** Department of Economics and Management, University of Pisa, Pisa, Italy

**Keywords:** Social mood, Economic sentiment, Istat, Sovereign bond yields, G41, E43, H63

## Abstract

By analysing the relationship between a new experimental daily index based on Twitter data (the Istat’s Social Mood on Economy Index—SMoEI) and the structure of Italian (and Spanish) sovereign interest rates, our work sheds new light on the great significance of the interconnections between economic sentiment and the Italian sovereign bond market. A placebo test performed on Spain introduces a possible extension of this linkage to the European market, highlighting the deep integration of financial markets within the European Monetary Union. Within a VAR and VECM framework with daily frequency data (2016–2022), we show that public shaping mechanisms play a role in the cost of debt financing. Our analysis emphasises the importance of economic sentiment when it comes to financial markets, putting the role of macroeconomic fundamentals in a different light. This result should be interpreted with caution, as updates to fundamentals can be affected by a time lag. In any case, recognising the importance of this index has at least two implications: on the one side, the SMoEI could represent a more responsive indicator for predicting investor sentiment; on the other side, media channels—as well as the European and national institutions—should gain relevance for their potential impact on collective sentiment, encouraging the importance of economic education and non-alarmist communication.

*If men define situations as real, they are real in their consequences*.

W. I. Thomas (American sociologist)

## Introduction

Social media, which have made social networks larger and more observable, have not only deeply influenced how we collect information about what is happening in the world but have also given anyone the possibility to spread his opinion worldwide. Focusing on Twitter, there exist millions of tweets related to financial markets. Since the contents of these tweets are generated by hundreds of millions of online users, they can mirror the collective opinion towards financial markets themselves. Behavioural economics has increasingly shown that psychological, cognitive, emotional, cultural and social factors may influence the decisions of individuals and institutions. The word *sentiment* is related to the possibility that investors’ expectations can diverge from those determined by rational behaviour. Macroeconomic and microeconomic fundamentals play a primary role in determining the performance of financial markets, but recently there seems to be more and more room for something beyond pure rationality which can be probably intercepted by social media. In other words, stock market behaviour can be analysed from the perspective of economic fundamentals but what investors think is what determines their decisions, and, in turn, what people think derives from how they feel, which is affected by their interactions with others (Nofsinger 2010).

Nowadays millions of Italians use social media platforms to keep up with the news, express their feelings and ideas, as well as to share or debate opinions on virtually every conceivable topic. The increasing popularity of these platforms has deeply affected the nature of the web, constantly evolving information. Over the last decade, Twitter has become one of the most famous (and reliable) social networking sites, depicting a primary source of real-time information.^1^ Among the different social media platforms, this platform has known widespread user adoption and rapid growth in communication volume. Its rapid diffusion, together with the fact that tweets often express users’ opinion on a specific topic of interest, has encouraged many researchers to exploit these features, developing two emerging approaches that can automatically detect text polarity within a huge amount of data, i.e. Opinion Mining (OM) and Sentiment Analysis (SA) (Giachanou and Crestani 2016). These two methodologies are often interpreted with the same meaning, but they deal with two different issues: OM analyses if a text contains an opinion, while SA assigns to this opinion a positive or negative sentiment (Tsytsarau and Palpanas 2012). In a nutshell, this platform can reveal collective opinions on several issues, such as the economy, therefore representing a unique opportunity for researchers interested in the study of consumers’ beliefs. This justifies the Italian National Institute of Statistics (Istat)’s idea to exploit social media sources and sentiment analysis techniques as a tool to measure the public mood in Italy, developing an experimental index, based on Twitter data: the Social Mood on Economy Index (SMoEI). At the time of writing, the daily time series of the index is available for the period 1st March 2016–30th June 2022, which also represents our period of analysis.

This new index represents only one aspect of our investigation, as our basic idea is based on its potential and reciprocal connections with the Italian sovereign debt market. In other words, is there—and what is the eventual nature of—a relationship between the economic sentiment that emerges among Italian Twitter users and the interest rates at different maturities in the Italian government debt market? In order to capture the financial interdependences among the Eurozone countries and to deepen what the Istat index is explaining (i.e. economic sentiment and/or sovereign default risk), we run a placebo exercise taking into consideration the sovereign bond yields of a similar European country (Spain). This comparison will be important to outline the main features of the SMoEI in relation to the Italian sovereign debt market within a currency area.

From this point of view, the SMoEI represents only one part of the analysis. Considering the sovereign bond markets of the countries of the European Monetary Union (EMU), it is preliminarily important to identify two different dynamics. In the years before and after the initial adoption of the single currency (1999), rapid convergence of the interest rates within the euro-area sovereign bond market can be generally observed. This behaviour has been considered fairly natural since there were no longer devaluation risks and the inflation was under the direct control of a single central bank, whose main objective was only to maintain price stability. Losing each country the ability to set its inflation rate independently, the EMU formally eliminates inflation risk on government debt (Alesina et al. 1992); while in the past expansionary fiscal policies were punished by financial markets with higher interest rates and, in some cases, with exchange rate depreciation, the latter aspect tends to vanish when a single currency begins to gather a relatively large group of countries (Faini 2006). After the bankruptcy of Lehman Brothers in September 2008, it begins to emerge a significant divergence of sovereign yields with debt markets experiencing severe financial stress since mid-2010. Since then, nominal interest rates begins to deeply diverge and financial markets become much more sensitive to macroeconomic fundamentals, especially after the Eurozone sovereign debt crisis. In particular, in the aftermath of the financial crisis, there has been a relevant turnaround in the intensity of the interest rate reaction to different determinants, mainly in the EMU (Afonso and Leal 2017). This growing sensitivity has largely concerned the high-yield economies in the European periphery (Beirne and Fratzscher 2013). In any case, if, at the peak of the crisis, the analysis of macroeconomic fundamentals had experienced great attention, afterwards the sovereign spreads returned to being driven by market sentiment (Alessi et al. 2019).

To the best of our knowledge, this is the first paper that empirically uses the SMoEI, trying to establish the nature of the linkage between the Italian (and Spanish) sovereign debt markets and collective sentiment. It is also one of the few papers that use a sentiment index to analyse the direct or reverse impact on a national financial market (government bonds) that goes beyond equity returns. The idea of applying a Vector Error Correction Model (VECM) in order to identify which market drives the price discovery process is also original since it has traditionally been used in relation to the sovereign and CDS debt market. In this regard, running the Johansen tests for cointegration, we check whether a cointegration relationship exists for the full range of maturities, preventing the error term in the long-run relationship from becoming larger and larger. By measuring the correction from the disequilibrium of the previous period, we try to establish which variable plays a fundamental role in the price discovery process. In this regard, our analysis wants to determine, among other things, if the SMoEI is able to show a connection with relatively advanced economic concepts. Our results shed new light on the great significance of the interconnections between economic sentiment and the Italian sovereign bond market. A placebo test performed on Spain also introduces a possible extension of this linkage to the European market, highlighting the deep integration of financial markets within the EMU.

The rest of the paper is structured as follows. Section 2 discusses from a theoretical perspective how economic literature deals with the problem of financial instability that is not correlated to fundamentals, and the main determinants of sovereign bond yields with a specific focus on the role played by social mood and economic sentiment. Section 3 introduces the data and describes the related methodology. Section 4 discusses the results, and Sect. 5 concludes.

## Social Mood and Financial Markets: Setting the Issue

Standard finance models are generally based on unemotional investors who always force capital market prices to equal the present value of expected future cash flows. These models tend to show considerable issues when they have to explain the significant changes in stock prices: according to the Efficient Market Hypothesis (EMH), stock market prices are mainly driven by news rather than present and past prices, and, since news is unpredictable, prices will follow a random walk pattern and cannot be predicted with great precision (Fama 1965; Qian and Rasheed 2007). In other words, the EMH postulates that asset prices should be based on all available information. A growing literature has critically examined the EMH (Malkiel 2003) and some recent research has shown that early signs of unpredictable news may be obtained from social media in order to forecast changes in many economic and financial indicators (Schumaker and Chen 2009). If it is true that the news affects stock market prices, the social mood also plays an equally important role. Behavioural finance has demonstrated that any financial choice is driven by emotion (Nofsinger 2010). As a consequence, public sentiment may drive stock market value, becoming important as any other quantitative measure. From a historical point of view, the idea of financial instability outside macroeconomic fundamentals can be traced back to Keynes (1936), who argues that markets can fluctuate wildly under the influence of investors’ sentiments (*animal spirits*).

Social media have significantly increased the possibility of (virtual) human interactions. As deemed by Prechter (1999), when emotion is shared by a society, the related level of optimism—or pessimism—influences financial decisions, potentially leading to a market-wide phenomenon. His ‘socionomic hypothesis’ underlines how human interactions favour the spread of emotions, characterizing how people eventually will act. Over time, several models have investigated the effect of social mood—at the beginning represented by newspapers and news coverage—on financial decisions. The advent of the first newspapers has been often considered the cause of the beginning of speculative bubbles:

*The news media are fundamental propagators of speculative price movements through their efforts to make the news interesting to their audience. They sometimes strive to enhance such interest by attaching news stories to price movements that the public has already observed, thereby enhancing the salience of these movements and focusing greater attention on them* (Shiller 2015).

Shiller (1981, 1984) already stressed the importance of social sentiment, observing that most investors do not have the necessary knowledge to understand the very volatile movements of financial markets: the process by which opinions are derived is interpreted as a social process, and the rate at which people are exposed to the changing attitudes and persuaded may be approximated by the infection rate. In this respect, the spread of opinions is directly compared to the spread of disease. Economic optimism—or pessimism—tends to quickly spread in financial markets, encountering much more difficulty and time to influence economic activity.

Based on these observations, behavioural finance has tried to develop alternative models based on two main assumptions; the first assumption is related to the finding that investors are inevitably subject to sentiment (De Long et al. 1990); the second assumption considers the bet against sentimental investors as costly and risky (Shleifer and Vishny 1997). As a consequence, rational investors—or arbitrageurs—do not aggressively force prices to fundamentals as the first kind of model suggests (Baker and Wurgler 2007). One of the first empirical attempts to deepen the interactions between news spread and stock prices is represented by the analysis of Cutler et al. (1989), but the theoretical fundamentals of the subsequent models about the effects of investor sentiment on stock market pricing have been introduced by De Long et al. (1990) (Tetlock 2007). Within an overlapping generations model, the latter authors underline that financial prices can diverge significantly from fundamental values even in the absence of relevant risks due to the unpredictability of noise trading.^2^ Noise traders are in fact characterised by random beliefs about the future compared to rational arbitrageurs, and their presence of noise traders is crucial in explaining why optimistic (pessimistic) sentiment may determine stock prices to exceed (go lower than) fundamental values for long periods:

*If noise traders today are pessimistic about an asset and have driven down its price, an arbitrageur buying this asset must recognize that in the near future noise traders might become even more pessimistic and drive the price down even further. If the arbitrageur has to liquidate before the price recovers, he suffers a loss. Fear of this loss should limit his original arbitrage position* (De Long et al. 1990).

This means that arbitrage does not remove the influence of noise because noise itself is able to determine risk. The importance of noise in financial markets had also been argued by Black in 1984:

*Noise makes trading in financial markets possible, and thus allows us to observe prices for financial assets. Noise causes markets to be somewhat inefficient, but often prevents us from taking advantage of inefficiencies. (…) Noise makes it very difficult to test either practical or academic theories about the way that financial or economic markets work. We are forced to act largely in the dark.*

More recently, Pedersen (2022) has introduced, borrowing some of the previous concepts, an interesting model of how investment decisions may spread through a social network and influence market behaviour and prices. Listening to all available information without paying much attention to their initial opinion, rational agents quickly become stubborn, determining a phenomenon that the author calls the *stubbornness of truth*: if no new relevant information emerges, they keep repeating their behaviours, characterising the market as rational in the long run. In any case, fanatic agents are also stubborn about their irrational personal views, affecting market prices. The consequent general trading activity is seen as an average of the reliance between rational and fanatic beliefs. Since the network component does not depend on fundamentals (investors learning via a social network react gradually to the news, disagreeing from rational views for long periods), bubbles, anti-bubbles and large price fluctuations can be observed in financial markets for irrational reasons.

Generally speaking, the main determinants of sovereign bond yields are three: credit risk, liquidity consideration, and changes in risk aversion. First of all, credit risk can be in turn divided into three different types of risk: the default risk, defined as the possibility that the issuer does not repay either the coupon or the principal; the credit spread risk, interpreted as the danger that the interest rate on a bond turns out to be too exiguous relative to an investment with a lower default risk; the downgrade risk, which reflects the chance of a downgrade by a credit rating agency. Generally, it is approximated by Credit Default Swaps (CDS) spreads (Favero 2013), and, from an empirical point of view, sovereign credit risk and risk aversion have been largely confirmed (Attinasi et al. 2009; Schwarz 2019). CDS is an alternative way to assess the default risk of a sovereign issuer, as it represents a financial swap agreement where the seller compensates the buyer in the event of a debt default or other credit event. To be more precise, the CDS price reflects both the expected loss of purchasing bonds, which depends on the probability of default and the recovery rate on the nominal value of the bonds, and the risk premium, where risk aversion and volatility play a fundamental role. Secondly, liquidity risk refers to the probability that a market is not characterized by a sufficient volume of buy and sell orders, as well as to the hazard that a large-scale transaction can strongly affect prices. Results related to this aspect are more complicated and controversial and liquidity considerations seem to become relevant only in the case of high market uncertainty (Liu 2014). Thirdly, risk aversion represents the propensity of investors to take the risk, which—during times of financial uncertainty—usually translates into a ‘flight’ to the risk-free sovereign market. In the European Union, as well-known, this role is played by Germany, whose bonds are perceived as safe-haven assets (Barrios et al. 2009). This general characterisation has to be framed within the European Monetary Union, which changes the relative importance of these three determinants. In particular, the loss of monetary sovereignty and the impossibility of devaluing the national exchange rate expose each member state to more a likely liquidity crisis and not just a solvency crisis as in the case to be characterized by a national lender-of-last-resort; in other words, from a theoretical point of view, the sovereign risk tends to be higher within a monetary union (Lemmen and Goodhart 1999). Empirically, global risk aversion is approximated using indices of stock market implied volatility*.* In particular, the VIX index is typically used as an index of the market sentiment of fear (Kilponen et al. 2015; Bernal et al. 2016).

More recently, behavioural finance started to introduce social mood as a new determinant for government bond yields. Although there are different perspectives, the dynamics of stock and bond markets are now considered to be influenced not only by financial variables, but also by sentiment and emotions that can be mined from news and social media (Blommestein et al. 2012). Analysing the tone of political communication during the sovereign debt crisis in Greece, Ireland and Portugal, Gade et al. (2013) find that negative or positive attitude has a contemporary effect on government bond interest rates. News pessimism in these countries, together with Italy and Spain, has been also deepened by Liu (2014) over the period 2009–2012, detecting a negative relationship between the spread of negative news and sovereign bond prices (“*no news is good news*”). Exploiting the EPU index, Bernal et al. (2016) argued that a higher level of uncertainty in one country determines a negative spillover effect in the Eurozone. According to Gotthelf and Uhl (2018), the sentiment of newspaper articles explains and forecasts changes in the term structure of US government bonds, since investor decision-making is becoming—due to growing uncertainty—increasingly dependent on news and sentiment. Exploiting an open-source database known as Global Database of Events, Language and Tone, Consoli et al. (2021) show that negative emotions mined from news significantly enhance the predictive power of government yield models.

In any case, it should be preliminarily noted that, from an empirical point of view, studying the relationship between social mood and financial markets first means constructing a proxy which is able to gather sentiment. The issue is no longer if sentiment is able to influence stock prices but rather how to gauge the social mood, quantifying its effects. From this point of view, depending on how this proxy is built, it is possible to distinguish three different strands of literature. The first strand uses market-based proxies, such as closed-end fund discounts (Lee et al. 1991; Swaminathan 1996; Neal and Wheatley 1998). One of the main drawbacks is connected with a natural problem of endogeneity, as these proxies are composed of the same financial indices they are supposed to measure. The second strand exploits surveys to create an investor sentiment index: using survey data on investor sentiment, Brown and Cliff (2005) provide evidence that sentiment affects asset valuation; Lemmen and Portniaguina (2006) take into consideration two surveys of consumer confidence conducted in the United States, showing that changes in the consumer confidence measure are strongly correlated with small stock returns. In these cases, the main risk is that these surveys are not adequately representative. Finally, the third and most popular strand of literature is based on news content, social media and exogenous events to derive a proper investor—or, more generally, social—sentiment. One important advantage of this kind of analysis is that search-based sentiment measures are available at a high frequency, representing a crucial feature especially when one has to deal with financial series. Using daily content from the *Wall Street Journal*, Tetlock (2007) shows that negative media sentiment forecasts downward pressure on equity market prices. Bollen et al. (2011) investigate if measurements of collective mood states derived from large-scale Twitter feeds are correlated to the value of the Dow Jones Industrial Average (DJIA) over time, demonstrating that the accuracy of DJIA predictions is improved when a specific public mood dimension is included. Siganos et al. (2014) propose to use an alternative measure of sentiment, based on status updates on Facebook, to examine its effect on trading behaviours. Their study is particularly important for at least three reasons: firstly, sentiment shows a positive contemporaneous correlation to stock returns; secondly, the authors find a precise direction of causality from sentiment to stock markets^3^; thirdly, negative sentiments increase trading volume and volatility. Da et al. (2015) construct a new measure of investor sentiment, using daily Internet searches related to household concerns about the economy, and show that their index predicts aggregate market returns and is significantly related to the transitory component of daily volatility and with VIX futures returns. In general, existing literature has tended to focus only on the impact of local investor sentiment on local market performance. On the contrary, Li et al. (2017) investigate the relationship between local daily happiness sentiment extracted from Twitter and stock returns of Chinese companies listed in the United States, highlighting the existence of a bi-directional linkage and, from a broader perspective, of a significant interdependence between online activities and stock market dynamics.^4^

## Data and Methodology

If applied on Twitter, SA is also called Twitter Sentiment Analysis (TSA). TSA is generally more complex than simple SA due to the intrinsic features of tweets, dealing with very short texts (maximum 140 characters), informal linguistic style, the presence of misspelt words, and the inattentive use of grammar (Martínez-Cámara et al. 2014).^5^ Within this context, Istat has developed an experimental index in order to evaluate the Italian collective mood about the economic situation. The underlying idea was to use social media messages as indicators of a collective sentiment in relation to a specific topic. In particular, this index, known as Social Mood on Economy Index (SMoEI), provides a high-frequency (daily) measure of the Italian sentiment on the state of the economy. From a methodological point of view, the procedure processes tweets containing at least one keyword belonging to a specific filter (i.e. a predefined set of relevant Italian words). The dictionary-based approach represents the most frequently used method in the literature (Li 2010). Generally speaking, filters should be able to capture relevant messages within a certain subject, eliminating at the same time off-topic ones: through a mapping algorithm, a computer program reads selected texts, classifying the keywords into different categories based on predefined dictionary classifications. Since 2016, the ‘Social Mood on Economy’ filter has been used on real-time samples of Italian tweets (57,000 per day). This filter, which represents the first level of the TSA, is based on 60 words or phrases that have been partially borrowed from the Italian Consumer Confidence Survey. A second level filter now exists, containing 115 keywords that have been subsequently added and that represent a strict subset of the previous filter. This refinement, based on a word embedding technique, makes it possible to identify economic tweets more likley, since the same word may have different meanings and be used in several contexts, but has the side effect of decreasing the volume of selected tweets (about 26,000 per day). In any case, as we will see, this reduction will not be so relevant thanks to a recent methodological improvement. None of the keywords included in the two filters has ever been made public. This deliberate decision has been justified by the intention to make the index less manipulable by misleading behaviours (i.e. Twitter bombing, which involves posting numerous tweets with similar content from multiple accounts with the goal of making a certain theme a trending topic).

The sentiment polarity classification problem is generally modelled as a two-way (positive/negative) or three-way (positive/negative/neutral) classification of a unit of text. Istat started with a two-way model, recently updating its methodology with a three-way categorisation. Formerly, tweets whose text matches at least one keyword belonging to the first level of the filter were collected. Each selected tweet was attached to sentiment scores, using an Italian lexicon (i.e. an Italian vocabulary whose lemmas are associated with precomputed positive and negative sentiment scores). Positive ($p$) and negative ($n$) sentiment scores of lemmas were constrained as follows:

1 $$\left\{\begin{array}{c}p\in [\mathrm{0,1}]\\ n\in [\mathrm{0,1}]\\ p+n\le 1\end{array}\right.$$

Therefore, the Italian lexicon mapped lemmas to points belonging to the sentiment triangle:
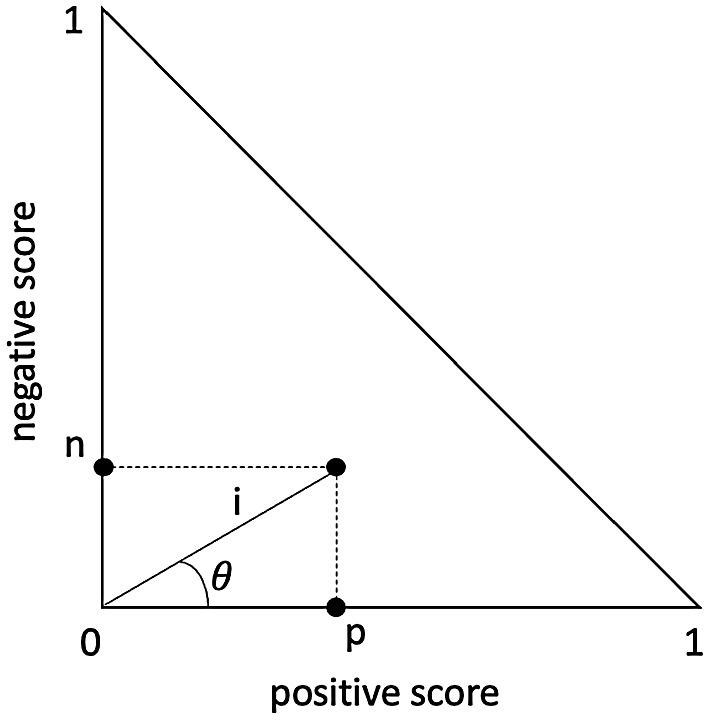


From ($p$, $n$) coordinates it was possible to estimate the polar coordinates ($i$, $\theta$) and derive polarity and intensity, obtaining a 4D sentiment space. Polarity ($\omega$) and intensity ($i$) could be defined in the following way,

2 $$\omega =1-4\theta /\pi \quad \omega \in [-1, 1]$$

3 $$i=\sqrt{{p}^{2}+{n}^{2}} \quad  i\in [0, 1]$$

For the sake of clarity, let’s suppose that the following Italian words have been extracted by a general Tweet: reform, bankruptcy, data, unemployment, youth, and increase. These keywords are characterised by a specific sentiment rating, whose average determines the related overall polarity and intensity (Table 1).

**Table 1:** An example of Twitter sentiment analysis in the first istat methodology

Words	$p$	$n$	$\omega$	$i$
Reform	0.125	0	1	0.125
Bankruptcy	0.021	0.375	− 0.929	0.376
Data	0.063	0.104	− 0.312	0.122
Unemployment	0	0.625	− 1	0.625
Youth	0.125	0	1	0.125
Increase	0.208	0.083	0.516	0.224
Average	0.090	0.198	− 0.455	0.217

Once sentiment scores ($p$, $n$, $\omega$, $i$) were available for all the tweets of a daily block, a K-means was used to cluster them into Positive, Negative and Neutral tweets. Lastly, the daily index value ($S$) was computed, depending on the distribution of tweets within the three different clusters:

4 $$S=\overline{{\omega  }_{i}}=\frac{{\sum }_{t}{i}_{t}{\omega }_{t}}{{\sum }_{t}{i}_{t}}=\frac{{\sum }_{t\in P}{i}_{t}{\omega }_{t}+{\sum }_{t\in N}{i}_{t} {\omega }_{t}}{{\sum }_{t}{i}_{t}} \quad  {\omega }_{t}\stackrel{\scriptscriptstyle\mathrm{def}}{=}0 \quad \forall t\in Neutral$$

The index could be then seen as the average of polarity ($\omega$) weighted by intensity ($i$), provided treat Neutral tweets as if their polarity were zero. In this way, the index was more resilient to misclassification and reduces day-to-day volatility. In other words, the daily index value is then derived as a central tendency measure of the score distribution of the tweets. This index is finally linearly transformed, also passing an outlier detection procedure in order to make it robust against possible contaminations by off-topic tweets.

Since the Italian lexicon did not provide any neutral sentiment score, clustering represented a crucial step for the classification of neutral tweets, excluding the latter from the index estimation. As anticipated, recently, the old Italian lexicon has been replaced in order to consider some neologisms linked to the Covid-19 pandemic, to remove terms that are not used in economic contexts, and to distinguish among positive, negative and neutral sentiment scores. The previous clustering operations are therefore no longer necessary, representing a fundamental improvement if we consider that the volume of filtered tweets has now been reduced due to the introduction of the second level of the filter. This makes the reduction from 57,000 to 26,000 tweets per day much less significant. The index calculation formula has remained unchanged.

As the index is based on daily values, it is not possible to identify the seasonal component with the standard TRAMO-SEATS methodology applied to the monthly or quarterly series. For the identification of the seasonal component in high-frequency series, Istat has followed an approach which applies modified exponential smoothing models (De Livera et al. 2010). The main problem deals with the presence of multiple seasonality, which implies the need for a two-step procedure: estimation of deterministic effects through the introduction of appropriate dummy variables and identification of the components of the linearised series. As a result of the seasonal adjustment, it is possible to isolate the linearised series of the index along with its trend and seasonal components.

**Fig. 1:**
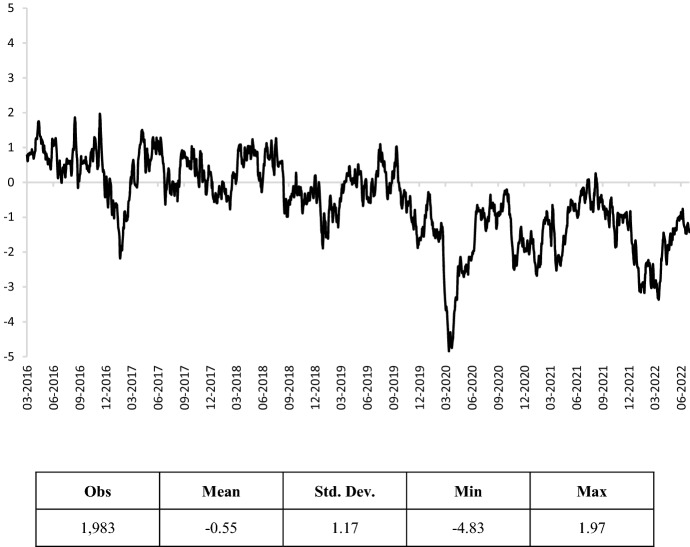
Social mood on economy index (daily trend). Source: own elaborations on Istat data

The end-of-week values of the SMoEI (daily trend) have been merged by the simple arithmetic mean, obtaining a series with 6 days per week. This adjustment has been introduced since bond markets are closed on those days. In order not to lose the information embodied in the weekend and to capture the potential changing economic sentiment, we also added to all the financial series an interpolation estimated as the mean between the Friday and the Monday interest rates. The SMoEI and its main descriptive statistics are depicted in Fig. 1. A preliminary analysis shows two interesting features. First of all, the social mood seems to react more to negative news than to positive ones, being included in a range between − 4.83 and 1.97; it tends also to be quite variable, being its standard deviation equal to 1.17. The second important feature is its significant persistence, which suggests that investors’ beliefs are not too volatile, and waves of optimism or pessimism seem to reinforce themselves.^6^

**Fig. 2:**
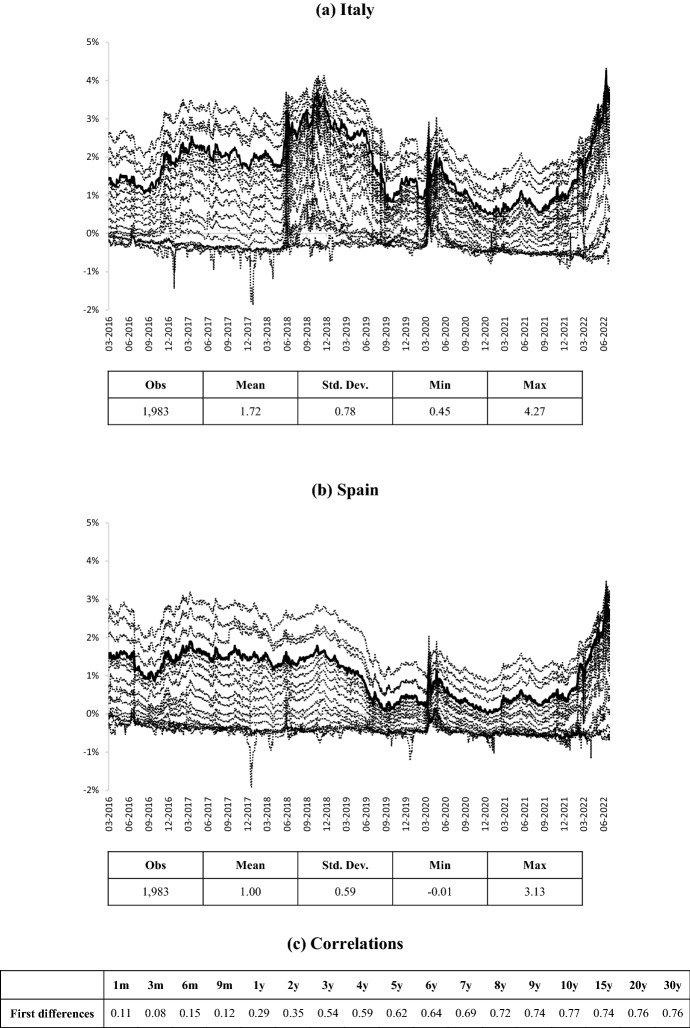
Sovereign bond interest rates at different maturities. The highlighted series and the main descriptive statistics in (**a** and **b**) correspond to the 10-year interest rate, which is considered the benchmark in the sovereign bond market. **c** Reports the correlations between the sovereign interest rates of the two countries at the related maturity; all correlations are significant at 1%. Source: own elaborations on Thomson Reuters data

Figure 2 reports the Italian (a) and Spanish (b) government interest rates at maturities from 1 month to 30 years, highlighting the 10-year yield, and the correlations (c) in first differences between the two series. In this regard, it is worth noting some aspects. First of all, the Italian 10-year sovereign yield has been on average higher than the Spanish one (respectively, 1.72% and 1.00%), with the spreads related to the minimum and maximum values equal to 0.46% and 1.14%; within this range, Italian interest rates have been characterised by a greater variability. Secondly, considering all the maturities, the difference between the shortest yield and the longest one has reduced significantly since the outbreak of the Covid-19 pandemic (March 2020). This narrow scale seems consistent with a collective sentiment that moves into negative territory. The average lower yields have been determined by the ECB’s extraordinary intervention (Pandemic Emergency Purchase Programme—PEPP) that has significantly eased tensions in the bond markets in the aftermath of the pandemic crisis (Carnazza and Liberati 2021). In any case, the war in Ukraine (February 2022) has changed an overall decreasing trend, pushing yields significantly upwards. Finally, the Italian and Spanish sovereign bond markets start to be highly correlated from the third year of maturity. As we discuss in the next paragraph, this result could explain why there will be a difference in the cointegration analysis between the SMoEI and the two financial markets.

Summing up, the variables of our interest are mainly two: on the one side, the SMoEI, and, on the other side, the Italian (and Spanish) sovereign bond yields.^7^ These two kinds of variables will represent the baseline specification of the VECM. As seen, government interest rates are also affected by other potential determinants. In this regard, Table 2 reports all the variables considered in our final empirical analysis.

**Table 2:** Variables under investigation

Variable	Frequency	Definition	Source
1	Daily	(Italian) Social mood on economy index (daily trend) (SMoEI)	Istat
2	Daily	Italian sovereign bond yield (IR)	Thomson Reuters
		Maturities from 1 month to 30 years	
3	Daily	Spanish sovereign bond yield (IR)	Thomson Reuters
		Maturities from 1 month to 30 years	
4	Daily	German sovereign bond yield (IR)	Thomson Reuters
		Maturities from 1 month to 30 years	
5	Daily	US Economic Policy Uncertainty index (EPU)	Baker et al. (2016)
6	Monthly	Italian Economic Policy Uncertainty index (EPU)	Baker et al. (2016)
7	Monthly	Spanish Economic Policy Uncertainty index (EPU)	Ghirelli et al. (2019)
8	Daily	FTSE MIB	Thomson Reuters
9	Daily	IBEX 35	Thomson Reuters
10	Daily	VDAX	Thomson Reuters
11	Daily	VIX	Thomson Reuters
12	Daily	Italian CDS spread (cds_spread)	Thomson Reuters
13	Daily	Spanish CDS spread (cds_spread)	Thomson Reuters
14	–	Italian government debt rating scale index (rating)	S&P, Moody's and Fitch
15	–	Spanish government debt rating scale index (rating)	S&P, Moody's and Fitch

**Fig. 3:**
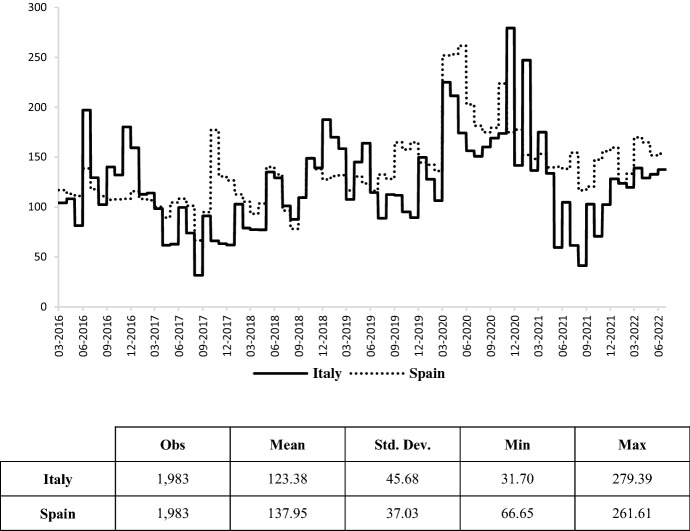
Economic policy uncertainty (EPU) index. The monthly index has been converted into daily data by repeating the same value during the related month. Source: own elaborations on Baker et al. (2016) and Ghirelli et al. (2019) data

The European Economic Policy Uncertainty (EPU) index represents our first control variable, and considers the coverage of economic uncertainty in major newspapers on a monthly basis.^8^ The Italian and Spanish policy-related economic uncertainty indices are based on *Corriere della Sera* and *La Stampa* for Italy and *El Mundo* and *El Pais* for Spain (Fig. 3). Newspaper articles to be matched have to contain the terms (in the native language) ‘uncertain’ or ‘uncertainty’, ‘economic’ or ‘economy’, and one or more policy-relevant terms.^9^ The EPU indices, originally developed by Baker et al. (2016), are probably the most popular in the field of economics derived from text-based (news) input. From an empirical point of view, the European and the United States indices have been found significant for understanding the transmission of risk from individual countries to the Eurozone bond market (Bernal et al. 2016).

**Fig. 4:**
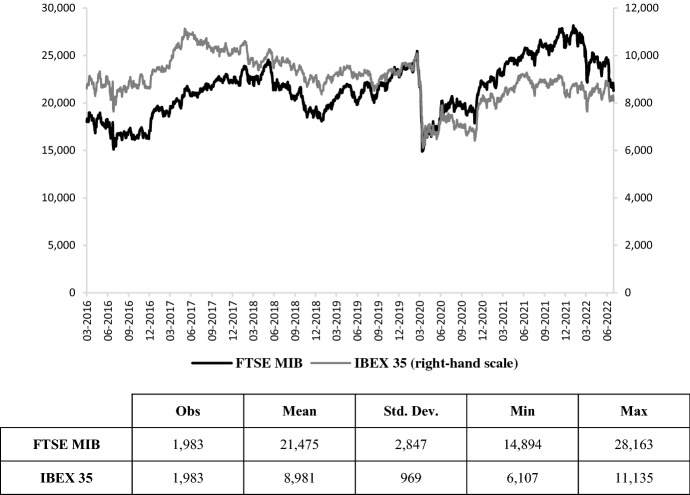
FTSE MIB and IBEX 35. Source: own elaborations on Thomson Reuters data

Secondly, we consider the equity return on the Italian (FTSE MIB) and Spanish (IBEX 35) stock markets (Fig. 4). The outbreak of Covid-19 has led to a fall in stock market indices, which have not yet managed to recover their previous value.

**Fig. 5:**
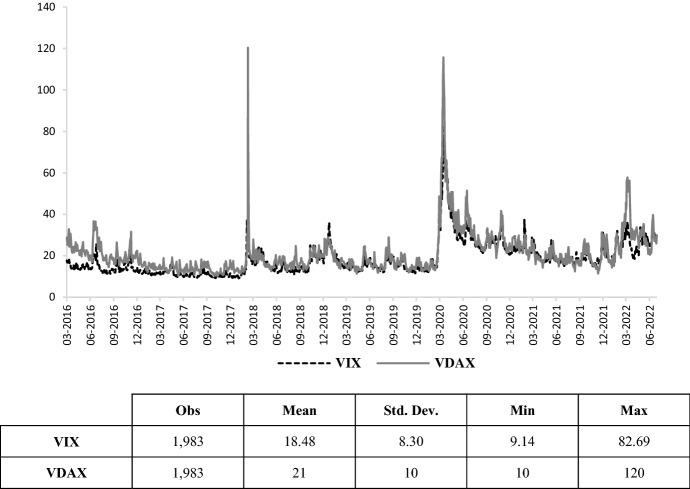
VDAX and VIX. Source: own elaborations on Thomson Reuters data

Thirdly, we include two alternative measures of market volatility. On the one side, the VIX, also known as the *fear index*, is a daily index of 30-day option-implied volatility in the S&P 500 (United States), representing one of the most popular measures of the stock market’s expectation of volatility. It is generally used to measure global uncertainty and aggregate risk aversion (Arghyrou and Kontonikas 2010). On the other side, the VDAX is analogous to the VIX, expressing the implied volatility of the DAX (Germany).^10^ Figure 5 displays the (very similar) evolution of these two indices during the period under consideration. The recent health crisis has led to greater volatility in the financial markets than the outbreak of war, while government yields have been characterised by the reverse reaction.

**Fig. 6:**
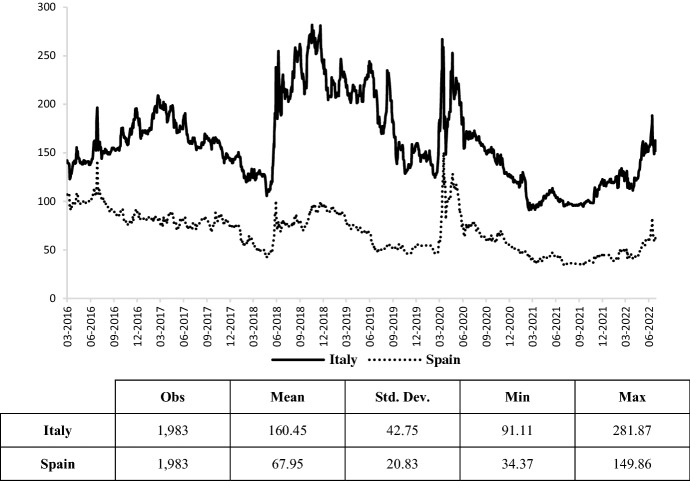
Italian and Spanish 10-year CDS spread. Source: own elaborations on Thomson Reuters data

Turbulence in financial markets does not seem to be influenced by the geographical proximity of the war conflict, as European CDS have also recently shown a smaller increase (Fig. 6). In this regard, CDS spreads between the Italian (or Spanish) and German 10-year CDS premiums have been then introduced in the empirical framework in order to isolate the default risk component of the two sovereign issuers.

**Fig. 7:**
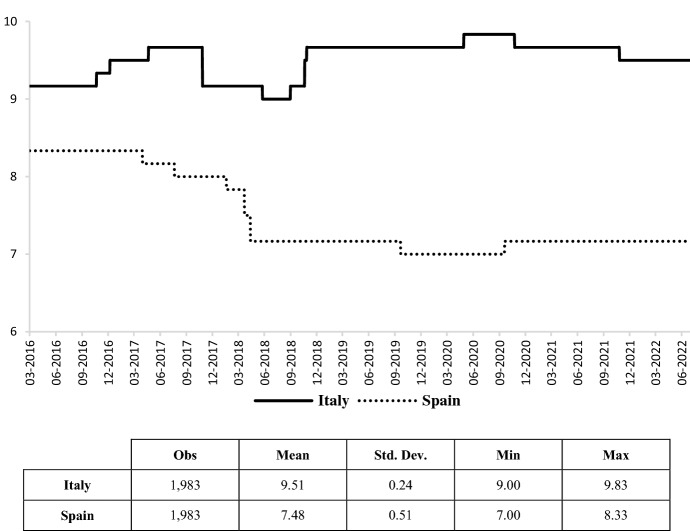
Government debt rating scale index. The index represents an arithmetic average of the assessments expressed by the three main rating agencies on the sustainability of Italian and Spanish public debt (the higher the score, the greater the probability of default). Source: own elaborations on Standard & Poor’s, Moody’s and Fitch Ratings data

Finally, credit rating agencies are among the most important vehicles for sovereign debt managers’ communication to the market, as they provide investors with analyses of the risks associated with debt securities. Given that credit rating agencies are meant to provide an assessment of the likelihood of default, we use ratings as a proxy of sovereign credit risk, by transforming the sovereign credit rating reviews (explicit credit ratings as well as credit outlooks) reported by Standard and Poor (S&P), Moody’s and Fitch into a discrete variable, which codifies the rating agency’s decisions according to a score classification (Fig. 7).^11^

Trended time series may determine significant issues in empirical econometrics due to the possibility of spurious regressions. This problem can be generally overcome, by differencing series until they become stationary and using them for regression analysis. In any case, by differencing both variables, we are differencing the error process, causing serious estimation difficulties, and we are implicitly determining a situation where the model may no longer give a unique long-run solution. This is the reason why we introduce cointegration analysis in order to investigate the relationship between sovereign bonds and Italian social mood, applying an empirical framework which usually takes into consideration two closely related markets (bonds and CDS) (Fontana and Scheicher 2016). Once the stationarity of the first difference of the variables of our interest has been verified, we run, based on Johansen’s method (1995), the tests for cointegration between the SMoEI and the Italian and Spanish sovereign bond yields at different maturities.^12^ This allows us to use a two-variable VECM, where the long-run equilibrium is given by a zero basis (i.e. no difference between SMoEI and interest rates). In this regard, we assume that the two variables evolve jointly over time and that in the long-term the two indicators should have the same value. This represents a hypothesis whose aim is to understand which variable is leading the other in the adjustment path (Coudert and Gex 2010). In particular, for each maturity $j$, the (baseline) model can be written in the following way:

5 $${\Delta SMoEI}_{t}={\beta }_{y0}+\sum_{i=1}^{n}{\beta }_{yi}{\Delta IR}_{t-i}+\sum_{i=1}^{n}{\delta }_{yi}{\Delta SMoEI}_{t-i}+{\lambda }_{1}\left({IR}_{t-1}-{\alpha }_{0}-{\alpha }_{1}{SMoEI}_{t-1}\right)+{\varepsilon }_{yt}$$

6 $${\Delta IR}_{t}={\beta }_{x0}+\sum_{i=1}^{n}{\beta }_{xi}{\Delta SMoEI}_{t-i}+\sum_{i=1}^{n}{\delta }_{xi}{\Delta IR}_{t-i}+{\lambda }_{2}\left({IR}_{t-1}-{\alpha }_{0}-{\alpha }_{1}{SMoEI}_{t-1}\right)+{\varepsilon }_{xt}$$

where ${SMoEI}_{t}={\alpha }_{0}+{\alpha }_{1}{IR}_{t}$ is the long-run cointegrating relationship between the two variables and ${\lambda }_{1}$ and ${\lambda }_{2}$ are the error correction parameters that measure how *SMoEI* and *IR* react to deviations from long-run equilibrium.^13^ In this model, we expect ${\lambda }_{1}<0$: if ${IR}_{t-1}$ is above its long-run value in relation to ${TSMoEI}_{t-1}$, then the error-correction term in parentheses is positive and this would lead, other things being constant, to downward movement in $TSMoEI$ in period $t$. ${\lambda }_{2}$ is also expected to be negative: since the long-run relationship between $TSMoEI$ and $IR$ is negative, then $IR$ would need to decrease in order to move toward equilibrium. In other words, since the cointegrating vector is characterised by negative coefficients, both long-run coefficients must be negative so that variables adjust to deviations from the equilibrium.

**Table 3:** VECM: baseline model

Maturity	Lag order (VAR)	Johansen tests for cointegration	Error correction term (EC)		Information share (S)	
			ΔSMoEI	ΔIR	ΔSMoEI (%)	ΔIR (%)
(a) Italy						
1-month	7	No				
3-month	5	No				
6-month	9	No				
9-month	6	No				
1-year	6	No				
2-year	6	No				
3-year	6	Yes	− 0.0089***	− 0.0013	100	0
4-year	6	Yes	− 0.0089***	− 0.0013	100	0
5-year	8	Yes	− 0.0091***	− 0.0007	100	0
6-year	6	Yes	− 0.0092***	− 0.0013	100	0
7-year	3	Yes	− 0.0100***	− 0.0011	100	0
8-year	2	Yes	− 0.0105***	− 0.0011	100	0
9-year	2	Yes	− 0.0106***	− 0.0012	100	0
10-year	2	Yes	− 0.0108***	− 0.0011	100	0
15-year	3	Yes	− 0.0104***	− 0.0012	100	0
20-year	2	Yes	− 0.0114***	− 0.0011	100	0
30-year	2	Yes	− 0.0120***	− 0.0008	100	0
(b) Spain						
1-month	3	No				
3-month	3	No				
6-month	3	No				
9-month	7	Yes	− 0.0104***	− 0.0011*	90.08	9.92
1-year	7	Yes	− 0.0102***	− 0.0009**	91.52	8.48
2-year	9	Yes	− 0.0100***	− 0.0015**	86.65	13.35
3-year	9	Yes	− 0.0100***	− 0.0013**	88.13	11.87
4-year	9	Yes	− 0.0102***	− 0.0017***	86.04	13.96
5-year	10	Yes	− 0.0100***	− 0.0013*	88.37	11.63
6-year	10	Yes	− 0.0106***	− 0.0016**	86.74	13.26
7-year	10	Yes	− 0.0113***	− 0.0019***	85.51	14.49
8-year	9	Yes	− 0.0122***	− 0.0023***	84.40	15.60
9-year	9	Yes	− 0.0123***	− 0.0024***	83.62	16.38
10-year	9	Yes	− 0.0121***	− 0.0022***	84.31	15.69
15-year	9	Yes	− 0.0125***	− 0.0022***	85.26	14.74
20-year	9	Yes	− 0.0136***	− 0.0026***	83.82	16.18
30-year	5	Yes	− 0.0137***	− 0.0021**	86.41	13.59

**Table 4:** VECM: exogenous controls

(a) Italy											
Maturity	ΔSMoEI										
	EC	ΔVDAX	Δi_de	ΔFTSE MIB	ΔEPU_it	Δrating_it	Δcds_spread_it	ΔIBEX 35	ΔEPU_es	Δcds_spread_es	ΔEPU_us
3-year	− 0.0089***			0.0001**	− 0.0006*		− 0.0020*			0.0043**	0.0000*
4-year	− 0.0089***			0.0001**	− 0.0006*		− 0.0021**			0.0045**	0.0000*
5-year	− 0.0089***			0.0001**	− 0.0006*		− 0.0020*			0.0044**	0.0000*
6-year	− 0.0093***			0.0001**	− 0.0006*		− 0.0020*			0.0043**	0.0000*
7-year	− 0.0100***			0.0001**	− 0.0006*		− 0.0018*			0.0040**	0.0000*
8-year	− 0.0106***			0.0001**	− 0.0006*		− 0.0018*			0.0041**	0.0000*
9-year	− 0.0106***			0.0001**	− 0.0006*		− 0.0018*			0.0041**	0.0000*
10-year	− 0.0108***			0.0001**	− 0.0006*		− 0.0019*			0.0041**	0.0000*
15-year	− 0.0108***			0.0001**	− 0.0006*		− 0.0018*			0.0040*	0.0000*
20-year	− 0.0114***			0.0001**	− 0.0006*		− 0.0018*			0.0040**	0.0000*
30-year	− 0.0120***			0.0001**	− 0.0006*		− 0.0018*			0.0040*	0.0000*

Maturity	ΔIR												Information share	
	EC	ΔVDAX	Δi_de	ΔFTSE MIB	ΔEPU_it	Δrating_it	Δcds_spread_it	ΔIBEX 35	ΔEPU_es	Δcds_spread_es		ΔEPU_us	ΔSMoEI (%)	ΔIR (%)
3-year	− 0.0004		0.8358***	− 0.0000***			0.0109***	0.0001***		0.0043***			100	0
4-year	− 0.0002		0.9000***	− 0.0000***			0.0106***	0.0001***		0.0038***			100	0
5-year	0.0000		0.8979***	− 0.0000***			0.0102***	0.0001***		0.0029***			100	0
6-year	− 0.0004		0.9360***	− 0.0000***			0.0109***	0.0001***		0.0016***			100	0
7-year	− 0.0001		0.9661***	− 0.0000***			0.0098***	0.0001***		0.0020***			100	0
8-year	− 0.0002		0.9640***	− 0.0000***			0.0100***	0.0001***		0.0011**			100	0
9-year	0.0001		0.9985***	− 0.0000***			0.0093***	0.0001***		0.0019***			100	0
10-year	0.0002		1.0110***	− 0.0000***			0.0090***	0.0001***		0.0015***			100	0
15-year	0.0001		0.8950***	− 0.0000***			0.0077***	0.0001***		0.0020***			100	0
20-year	0.0004		0.8799***	− 0.0000***			0.0076***	0.0000***		0.0013***			100	0
30-year	0.0004		0.8348***	− 0.0000***			0.0063***	0.0000*		0.0012***			100	0

(b) Spain											
Maturity	ΔSMoEI										
	EC	ΔVDAX	Δi_de	ΔFTSE MIB	ΔEPU_it	Δcds_spread_it	ΔIBEX 35	ΔEPU_es	Δrating_es	Δcds_spread_es	ΔEPU_us
9-month	− 0.0103***			0.0001**	− 0.0006*	− 0.0021**				0.0042**	0.0000*
1-year	− 0.0103***			0.0001**	–	− 0.0021**				0.0047**	
2-year	− 0.0100***			0.0001**	− 0.0006*	− 0.0021**				0.0046**	0.0000*
3-year	− 0.0100***			0.0001**	− 0.0006*	− 0.0021**				0.0044**	
4-year	− 0.0103***			0.0001**	− 0.0006*	− 0.0021**				0.0044**	0.0000*
5-year	− 0.0099***			0.0001**	− 0.0006*	− 0.0021**				0.0046**	0.0000*
6-year	− 0.0107***			0.0001**	− 0.0006*	− 0.0021**				0.0044**	0.0000*
7-year	− 0.0115***			0.0001**	− 0.0006*	− 0.0022**				0.0044**	0.0000*
8-year	− 0.0125***			0.0001**	− 0.0006*	− 0.0023**				0.0046**	0.0000*
9-year	− 0.0126***			0.0001**	− 0.0006*	− 0.0022**				0.0045**	0.0000*
10-year	− 0.0125***			0.0001**	− 0.0006*	− 0.0023**				0.0046**	0.0000*
15-year	− 0.0128***			0.0001**	− 0.0006*	− 0.0021**				0.0044**	0.0000*
20-year	− 0.0141***			0.0001**	− 0.0006*	− 0.0021**				0.0044**	0.0000*
30-year	− 0.0140***			0.0001**	− 0.0006*	− 0.0021**				0.0043**	0.0000*

Maturity	ΔIR												ΔSMoEI (%)	ΔIR (%)
	EC	ΔVDAX	Δi_de	ΔFTSE MIB		ΔEPU_it	Δcds_spread_it	ΔIBEX 35	ΔEPU_es	Δrating_es	Δcds_spread_es	ΔEPU_us	Information share	
9-month	− 0.0009	− 0.0004*	0.1606***	0.0000**			− 0.0008***	0.0000***			0.0032***	0.0000*	100	0
1-year	− 0.0006		0.3017***	0.0000*			0.0005***				0.0010**		100	0
2-year	− 0.0007		0.6210***				0.0009***	0.0000**			0.0031***		100	0
3-year	− 0.0003		0.7304***				0.0019***				0.0027***	0.0000**	100	0
4-year	− 0.0007*	− 0.0003*	0.7770***				0.0018***				0.0029***	0.0000**	93.87	6.13
5-year	0.0000	− 0.0003*	0.8233***				0.0018***				0.0043***	0.0000*	100	0
6-year	− 0.0004	− 0.0003*	0.8488***				0.0018***	0.0000*			0.0045***	0.0000*	100	0
7-year	− 0.0006	−0.0005***	0.8666***	0.0000*			0.0018***				0.0049***		100	0
8-year	− 0.0007		0.8850***				0.0022***	0.0000**			0.0046***		100	0
9-year	− 0.0007		0.9009***	0.0000**			0.0021***			0.0676	0.0045***		100	0
10-year	− 0.0004		0.9162***	0.0000*			0.0022***	0.0000*		0.0745	0.0050***		100	0
15-year	− 0.0001		0.8536***	0.0000			0.0019***			0.0741			100	0
20-year	− 0.0006		0.8400***				0.0021***	0.0000**		0.0737	0.0049***		100	0
30-year	− 0.0002		0.8498***				0.0020***	0.0000**		0.0791	0.0048***		100	0

According to this methodology, if ${\lambda }_{1}$ is statistically significant and ${\lambda }_{2}$ is not, the price discovery process is led by the SMoEI. The opposite situation would imply that the adjustment towards the long-run equilibrium of the two variables is led by the government interest rates. It may happen that both ${\lambda }_{1}$ and ${\lambda }_{2}$ are statistically significant, which means that both the social mood and the bond market play a role in the price discovering process. In this case, regardless of the signs of the coefficients, normalisation can be used (Schwarz and Szakmary 1994) to get the information share driven by each series. The information share ${S}_{k}$ is then defined in the following way:

7 $${S}_{k}=0<\frac{\left|{\lambda }_{k}\right|}{\left|{\lambda }_{1}\right|+\left|{\lambda }_{2}\right|}<1 \quad  k=1, 2$$

If ${S}_{1}>0.5$, the price discovery process is mainly driven by the social mood, while the reverse (*i.e.* the price discovery process is led by the government bond market) is true for ${S}_{1}<0.5$. An opposite interpretation occurs when looking at ${S}_{2}$.

In order to preserve the cointegration relationship between the two variables, we estimate a Vector Autoregressive (VAR) model in levels for the maturities where this relationship has been found significant (Auerbach and Gorodnichenko 2012; Caggiano et al. 2015; Kilian and Lütkepohl 2017); in the other (few) cases, we consider their first difference. These formulations are based on the underlying VAR, complete with all the exogenous controls, of the VECM specification introduced in Eqs. (5) and (6), where the first difference specification ($\Delta$) applies only to the latter cases. Results have been obtained by using HAC robust standard errors, that take into account heteroskedasticity and autocorrelation. One of the good features of these models is that they allow us to check the direction, if any, of (Granger) causality (Granger 1969). In any case, this kind of causality should be interpreted carefully since it refers only to the ability of one variable to predict the other in temporal terms and not in causal ones.

## Main Results

The VECM framework plays a fundamental role in our aim to investigate the existence—and possible nature—of a relationship between a national indicator of economic sentiment, represented by the SMoEI, and the Italian (and Spanish) sovereign debt markets. The Spanish case has been introduced in order to capture potential financial interdependences between two Eurozone countries, and to better understand what the Istat index is representing (*i.e.* economic sentiment and/or sovereign default risk). The related VAR models are thereafter important to better interpret the previous results.

Table 3 introduces the baseline results of the VECM analysis in relation to Italy (a) and Spain (b). Each maturity is associated with a certain lag order of the underlying VAR model, which has been taken into account to test for the presence of cointegration (Johansen tests). The two countries are characterised by a different start date of the cointegrating relationship between the two variables: respectively, 3 years for Italy, and 9 months for Spain.^14^ The linkage between the SMoEI and the Spanish market does not seem surprising. Studying the effect of news and emotions on Italian and Spanish government bond yields, Consoli et al. (2021) recently confirmed that news plays an important role in determining the related changes, stressing an interesting aspect: Italy is mainly concerned about national economic context, while Spain mainly looks at international events. According to the authors, there seems to emerge an emotive spillover effect: emotions generated by the Italian political turmoil propagate to the Spanish news affecting the neighbourhood market. This result may explain why an Italian sentiment indicator is connected to the dynamics of another country’s sovereign bond market. On the other hand, it cannot be excluded the possibility that this index also captures Italian concerns about the economic situation in Spain. In any case, it should be kept in mind that the sovereign bond markets of Eurozone countries are deeply interconnected and financial turmoil can easily spread. In this regard, the Italian and Spanish CDS spreads, which are supposed to seize the perception of country risk by financial operators, are highly correlated (0.72 in terms of first differences). This may mean that national economic sentiment has a high probability of capturing the same dynamics in similar European countries.

The VECM analysis has been performed only if a long-run cointegrating relationship exists between each maturity and the SMoEI. The ‘$\Delta SMoEI$’ column refers to Eq. (5), ‘$\Delta IR$’ to Eq. (6), and the error correction term to the related ${\lambda }_{1}$ and ${\lambda }_{2}$ parameters (Table 3). From this point of view, the Italian sovereign bond market is characterised by an adjustment process towards the long-run equilibrium that derives from the economic sentiment. In other words, according to our methodology, the price discovery process is exclusively driven by the SMoEI, with longer maturities adapting faster than shorter ones. Since ${\lambda }_{2}$ is never significant, the information share ${S}_{1}$ is always equal to 100%. The case of Spain is slightly different since the error correction parameter associated with the sovereign bond yields (${\lambda }_{2}$) has been found significant. This implies that it cannot now be ruled out that the price discovery process is also driven by the interest rate. Since ${\lambda }_{1}$ and ${\lambda }_{2}$ are both significant, the information share $S$ becomes crucial to understand which variable is leading the other: once again, the price discovery process results mainly led by the SMoEI, especially for shorter maturities (i.e. 90.52% at 1-year maturity).

Some might argue that the Istat index influences the dynamics of interest rates only because it reflects concerns about the macroeconomic fundamentals of a country. For this reason, we replicate the previous analysis by adding all the exogenous controls in an attempt to intercept this type of concern. In this regard, Table 4 is organised as Table 3, with the exception of omitting the specification of the lag order and the presence of the cointegrating relationship for the previous maturities. In relation to Italy (a), the price discovery process continues to be driven by the SMoEI *λ*_1_ shows similar values to the baseline model), and the parameter *λ*_2_ remains not significant. In other words, all control variables do not change the nature of the interrelationships between national economic sentiment and the Italian sovereign bond market. Focusing on the signs of the estimated coefficients associated with exogenous controls, the SMoEI is negatively affected by Italian economic uncertainty (the influence of the US high-frequency index is significant but very low) and the Italian CDS spread: an increase in domestic economic policy uncertainty and/or CDS spread determines a worsening of the Italian economic sentiment. On the other hand, the Spanish CDS spread and, to a reduced extent, the equity return on the Italian stock market (FTSE MIB) are positively interconnected with the SMoEI. As regards the Italian sovereign bond market, domestic interest rates are positively linked to the German yields, and the Italian and Spanish CDS spread. The coefficients associated with the two stock markets (FTSE MIB and IBEX 35) are significant but not so relevant in their amounts. Regarding Spain, the baseline result is even more robust: the price discovery process is driven by the SMoEI with higher information shares.

In order to corroborate and deepen the previous results, Table 5 presents the (Granger) causality between the Italian and Spanish sovereign interest rates and the SMoEI within a VAR framework with all the exogenous variables. Far from considering it a hint of causation, we exploit its meaning in temporal terms: Granger causality refers to timing anticipation, not causation. Focusing on the maturities where the cointegrating relationship has been found significant, the SMoEI anticipates changes in Italian government bond yields. On the contrary, the Istat index impact on the Spanish sovereign bond market is more controversial: in the short term, there is no (Granger) causal connection between the two variables despite the fact that the long-run cointegration relationship has shown that the price discovery process is however mainly driven by the SMoEI.

**Table 5:** Granger causality

Maturity	Italy	Levels		First differences		Spain	Levels		First differences	
	Lag order (VAR)	IR—> SMoEI	SMoEI—> IR	IR—> SMoEI	SMoEI—> IR	Lag order (VAR)	IR—> SMoEI	SMoEI—> IR	IR—> SMoEI	SMoEI—> IR
1-month	6			Yes***	No	6			No	No
3-month	4			Yes**	Yes***	4			No	No
6-month	8			No	No	8			No	No
9-month	5			No	No	6	Yes*	Yes*		
1-year	5			No	No	6	Yes**	No		
2-year	5			No	No	6	No	Yes*		
3-year	6	No	Yes*			6	No	Yes*		
4-year	6	No	Yes*			6	No	No		
5-year	8	No	Yes*			8	No	No		
6-year	6	No	Yes*			6	No	No		
7-year	3	No	Yes**			3	No	No		
8-year	2	No	Yes**			2	No	No		
9-year	2	No	Yes**			2	No	No		
10-year	2	Yes*	Yes*			2	No	No		
15-year	3	No	Yes*			3	No	No		
20-year	2	No	Yes*			2	No	No		
30-year	2	No	No			2	No	No		

The sign—> denotes the direction of the Granger causality (i.e. ‘*IR—*> *SMoEI*’ means that *IR* Granger causes *SMoEI*)

## Concluding Remarks

In the last fifteen years, financial markets have become much more sensitive. Especially after the Eurozone sovereign debt crisis and in the European periphery, macroeconomic fundamentals have gained great attention—a deteriorating fiscal position began to be gradually interpreted by investors as a sign of increased risk of sovereign default – but not as much as market sentiment (Alessi et al. 2019). In this regard, Caravaggio and Carnazza (2022) have recently introduced critical issues regarding the negative connotations often associated with public finance variables. From this point of view, it is possible that the progressive tightening of the European fiscal rules and the tale behind their importance could have exacerbated speculative dynamics at times of the greatest financial tensions. Gade et al. (2013) already stressed the negative influence that political miscommunication had during the European sovereign debt crisis, highlighting that pessimistic viewpoints may have added uncertainty to market perceptions about the financial sustainability of highly indebted countries. On the other side, the European institutions have also been found effective in moderating the negative effects of the rise of populist forces in Italy on financial markets (Balduzzi et al. 2020), which further emphasises the importance of the supranational context in affecting the sovereign bond yields of the most indebted countries. By analysing within a VAR and VECM framework the relationship between a new experimental index based on Twitter data (the Istat’s Social Mood on Economy Index) and the structure of Italian (and Spanish) sovereign interest rates, our work underlines the relevance of the interconnections between economic sentiment and sovereign bond markets. Public shaping mechanisms seem to play a relevant role in the cost of debt financing. This result should be interpreted with care, as updates related to fundamentals can be affected by a time lag, anticipated and incorporated by media pessimism (Liu 2014). This is a limitation of our analysis, besides the fact that we deal with daily data, thus losing relevant intraday information. Moreover, the results for Spain open up interesting questions about the interconnections between economic sentiment and a European sovereign debt market that appears deeply integrated. This could represent a continuation of the present work, looking for a European sentiment index and/or for country-specific sentiment index. Potential sentiment spillover effects may then play a role in increasing or decreasing financial turbulence within the EMU. In any case, our analysis emphasises the importance of economic sentiment when it comes to financial markets, putting the role of the macroeconomic fundamentals in a different light.

We see at least two implications. First of all, when considering the sovereign debt market and the related financing costs, it might be useful to consider a more reactive indicator, forecasting investor sentiment with the spread of negative news. Secondly, media channels—as well as the European and national institutions—should take into consideration their potential impact on collective sentiment. Our results encourage the importance of economic education and non-alarmist communication. Taking as an example a bank run, Merton (1948) theorised the idea of a self-fulfilling expectation: “*the prophecy of collapse led to its own fulfilment*”. Given the importance of collective sentiment, this concept, and our findings, could be useful in dealing with macroeconomic and financial turbulence.

## Footnotes

1 It should be noted that Twitter users are more likely than the general public to have a college degree (Wojcik and Hughes 2019). Compared to other social networks, this makes Twitter a more reliable and credible social media.

2 Summers (1986) has called this phenomenon ‘noise trading’, which is mostly related to financial speculation based on the past movements of price changes rather than based on economic fundamentals.

3 In any case, as the same authors underline, their results on causality have to be interpreted with appropriate caution mainly for two reasons. On the one side, the relation under investigation can be subjected to reverse causality, as good market performance could create positive feelings (Brown and Cliff 2004). Heimer and Simon (2015) also suggest that traders with good performance are more likely to communicate about their trading activity on social media. On the other side, several events might affect both sentiment and stock returns.

4 The happiness data is derived from the website (http://hedonometer.org/index.html), which is based on the results of the natural language processing technique on around 10% of all tweets using the English language, scoring the magnitude of happiness of selected words that appeared on Twitter, i.e. laughter, love, successful, winning, healthy and celebration.

5 For more information about the state-of-the-art in TSA, see Zimbra et al. (2018) and Carvalo and Plastino (2020).

6 The autocorrelation function (ACF) is positive and significant at 1% until the 706 lag (i.e. lag 30 is equal to 0.72, lag 60–0.53, lag 90–0.40, and lag 120–0.33).

7 The German sovereign bond market will be also taken into account to control for the level of interest rates in Europe, consistent with the corresponding maturity year.

8 In order not to miss any information on high-frequency political uncertainty, we also include the daily US EPU index.

9 The Italian EPU index has been developed by Baker et al. (2016). Following the newspaper-based methods contained in the previous article, Ghirelli et al. (2019) have estimated the Spanish EPU index.

10 We consider the VDAX as the main control variable for market volatility. Results with the VIX are robust and available upon request.

11 According to the three credit rating agencies, financial activities can be divided into two categories: investment grade and speculative assets. Each category can be in turn decomposed into four other classes. It is possible to create a ranking, where the highest score (1) is assigned to the safest financial investment, while the lowest one (21) to the most speculative asset. The credit outlook has also been taken into account by attributing to ‘positive’ a score of − 0.5, to ‘neutral’ of 0 and to ‘negative’ of + 0.5.

12 We run the ADF (Dickey and Fuller 1979, 1981), PP (Phillips and Perron 1988) and KPSS (Kwiatkowski et al. 1992) tests in order to know the order of integration. Results are available upon request.

13 According to our hypothesis, ${IR}_{t-1}-{\alpha }_{0}-{\alpha }_{1}{TSMoEI}_{t-1}=0$. The baseline results will be presented in Table 3, while Table 4 will include all the other variables (EPU indices, VIX, CDS spreads and ratings) as exogenous controls.

14 It should be noted that, as seen in the previous paragraph, Italian and Spanish government interest rates show precisely a high correlation starting from the third of maturity (Fig. 2c).
